# Using flow cytometry for mitochondrial assays

**DOI:** 10.1016/j.mex.2020.100938

**Published:** 2020-05-28

**Authors:** Lauar de Brito Monteiro, Gustavo Gastão Davanzo, Cristhiane Favero de Aguiar, Pedro M.M. Moraes-Vieira

**Affiliations:** a Division of Metabolism, Experimental Medicine Research Cluster (EMRC), and Laboratory of Immunometabolism, Department of Genetics, Evolution, Microbiology and Immunology, Institute of Biology, University of Campinas, SP, Brazil

**Keywords:** Immunometabolism, Mitochondrial function, Mitochondrial membrane potential, Metabolic reprogramming

## Abstract

The understanding of how different cell types adapt their metabolism in the face of challenges has been attracting the attention of researchers for many years. Recently, immunologists also started to focus on how the metabolism of immune cells can impact the way that immunity drives its responses. The presence of a pathogen or damage in a tissue changes severely the way that the immune cells need to respond. When activated, immune cells usually shift their metabolism from a high energy demanding status using mitochondria respiration to a glycolytic based rapid ATP production. The diminished amount of respiration leads to changes in the mitochondrial membrane potential and, consequently, generation of reactive oxygen species. Here, we show how flow cytometry can be used to track changes in mitochondrial mass, membrane potential and superoxide (ROS) production in live immune cells.

● This protocol suggests a quick way of evaluating mitochondrial fitness using flow cytometry. We propose using the probes MitoTraker Green and MitoTracker Red/ MitoSOX at the same time. This way, it is possible to evaluate different parameters of mitochondrial biology in living cells.

● Flow cytometry is a highly used tool by immunologists. With the advances of studies focusing on the metabolism of immune cells, a simplified application of flow cytometry for mitochondrial studies and screenings is a helpful clarifying method for immunology.

## Graphical abstract

**Figure:**
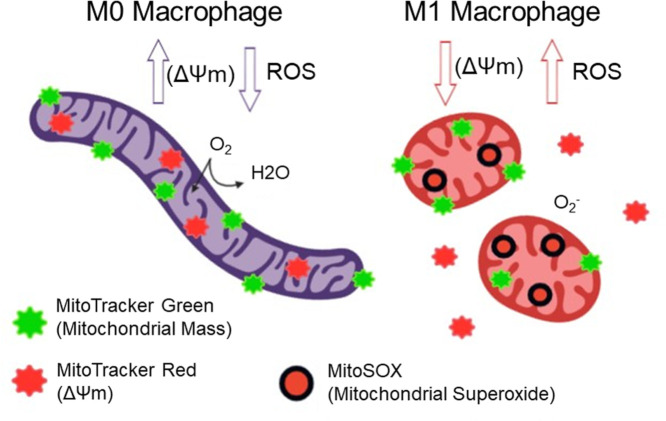

Specifications Table

**Table:** 

Subject Area	Immunology and Microbiology
More specific subject area	Immunometabolism
Method name	Flow cytometry – Mitochondrial staining
Name and reference of original method	Cell staining with mitochondrial probes (MitoTracker Green, Red and MitoSOX) and acquisition in flow cytometer has been published by Hagai Rottenberg and ShaolongWu. (1998). “Quantitative assay by flow cytometry of the mitochondrial membrane potential in intact cells. Biochimica et Biophysica Acta (BBA) - Molecular Cell Research 1404 (3): 393–404.” “Kauffman ME, Kauffman MK, Traore K, Zhu H, Trush MA, Jia Z, Li YR. MitoSOX-Based Flow Cytometry for Detecting Mitochondrial ROS. React Oxyg Species (Apex). 2016;2(5):361–370. doi: 10.20455/ros.2016.865.”
	We take advantage of flow cytometry, which is a simple and fast methodology, to evaluate various parameters regarding mitochondrial biology upon physiological and pathological conditions.
Resource availability	*n/a*

## Introduction

The metabolism of a cell is an intriguing dynamic workflow of metabolites and enzymes that adapt to the needs of each cell in a certain environment. At the end of the day, the main goal of the cell is to produce enough ATP to supply autocrine needs and paracrine or endocrine secretion of different types of molecules and the synthesis of biomolecules. In the last 200 years, researchers around the globe have been studying how different energy sources, i.e. as glucose, fatty acids, and amino acids can be used by different cell types to drive surveillance, proliferation, secretion and all the other physiological and even pathological functions. It has become clear that cells are in general plastic, adapting the metabolism according to the source of energy available or the cell function that has to be done. Using the expertise that biochemists built during the 19^th^ and 20^th^ century, immunologists started to investigate how immune cells adapt their metabolism during the challenges of a pathogen or damage challenge, creating a new field named immunometabolism.

The importance of metabolism for immune cells was revised by our group and others recently [1,2]. In the context of the immune system, it is well accepted that proliferative cells, as anti-inflammatory macrophages (M2), use the oxidative metabolism to produce ATP. Most of the ATP generated in a cell is done by the collaborative wok of the Krebs cycle and electron transport chain (ETC) into the mitochondria. The full oxidation of molecules to CO_2_ generates a pH gradient and a transmembrane potential through the pumping of protons by the complexes I, III and IV of the ETC. This gradient of protons is essential for ATP synthase to convert ADP and phosphate into ATP [1]. However, under pro-inflammatory activation, there is a shift from OXPHOS to glycolysis, leading to hyperpolarization of the inner mitochondrial membrane. This hyperpolarization generates mitochondrial superoxide - one type of reactive oxygen species (mtROS), an essential molecule for the killing of pathogens inside the phagosomes in macrophages [2]. Besides that, intracellular ROS has an important role in cell signaling, including the activation of keys inflammatory pathways, such as NF-κB, ERK and PI3K-Akt [3].

In the last 20 years the development of new technologies allowed researchers to better understand how mitochondria changes during different stimuli. Here, we describe how mitochondrial dyes can be used to track mitochondrial content (as MitoTracker Green – Thermo Scientific), mitochondrial membrane potential (MitoTracker Red - Thermo Scientific) or mitochondrial superoxide (MitoSox - Thermo Scientific) using flow cytometry. In this work, we use bone-marrow derived macrophages (BMDMs) to show how flow cytometry can be used to understand mitochondrial profile of live cells.

## Method details

### Macrophage differentiation

Male C57BL/6 mice, 8 weeks of age were used for all experiments. We collected the bone marrow of the femur and tibia using a syringe and flushed the cells into a Falcon tube with PBS + 2% FCS. Cell suspensions was then filtered through a cell strainer and centrifuged. Red blood cells were lysed and 5.10^6^ cells/mL were incubated at 37 °C with 5% CO_2_ for 6 days in the presence of 20 ng/mL of recombinant M-CSF. On the seventh day, macrophages were stimulated with either: Vehicle (PBS), LPS (100 ng/mL) or IFN- γ (20 ng/mL) +LPS (100 ng/mL). Mouse studies were conducted in accordance with federal guidelines. Animal use was approved by the Ethics Committee on Animal Use (CEUA – Protocol number: 4493–1). Mice were kept at SPF animal facility in the department of Genetics, Evolution, Microbiology and Immunology of the University of Campinas.

### MitoTracker green and mitotracker red staining for functionality assay

MitoTracker Green details:

­ Stock solution (reconstituted in DMSO): 1 mM
­ Working concentration: 20–200 nM

MitoTracker Red details:

- Stock solution (reconstituted in DMSO): 1 mM
- Working concentration: 25–500 nM

#### Preparation of probes

First, we calculate how many samples will be stained with the complete mix. We use 50 µL of probes mix per well. We use PBS + 2% FCS to dilute the probes and stain the cells.

For this experiment, we used the following panel to stain the macrophages (Table 1).

**Table 1:** Flow cytometry panel for mitochondrial parameters in macrophages.

Target/Probe	Fluorochrome	Clone/ Cat. number
Live/Dead	V500 - Zombie Aqua™ Excitation⁄Emission (nm): 367/526	LIVE/DEAD™ Fixable Aqua Dead Cell Stain Kit, for 405 nm excitation ThermoFisher Scientific Catalog number: L34957
Anti-mouse F4/80	Brilliant Violet 421™ Excitation⁄Emission (nm): 405/21	Clone: BM8 Isotype: Rat IgG2a, κ Biolegend Catalog number: 123,137
MitoTracker^Ⓡ^ Green	FITC – Green Excitation⁄Emission (nm): 490/516	MitoTracker™ Green FM - Special Packaging Invitrogen™ Catalog number: M7514
MitoTracker^Ⓡ^ Red	PE – Red Excitation⁄Emission (nm): 579/599	MitoTracker™ Red CMXRos - Special Packaging Invitrogen™ Catalog number: M7512

For macrophage surface staining and Live/ Dead staining, we diluted the anti-F4/80 antibody 1/200 and the Live/Dead probe was diluted 1/500.

From the stock solution of MitoTracker Green, we suggest diluting 10000x and keep a final concentration of 100 nM. This can be achieved by diluting the stock solution 1/100 in PBS and then diluting 1/100 again into the final mix.

For MitoTracker Red, we dilute the stock solution once (1/100) in PBS and then again 1/50 into the final mix for a final concentration of 50 nM.

We also separated one well for our unstained control and two wells for our MitoTracker Green and MitoTracker Red FMOs (fluorescence minus one). Each FMO control contained all the dyes, except either MitoTracker Green or Red.

Pre-warm the mix (37 °C).

#### Cell staining

Wash the cells with cold PBS 1x. We used a 48-well culture plate with 3.5 × 10^5^ cells.

If you are working with adherent cells, gently scrape the cells from the plate with a cell scraper.

Transfer the cells to a 96-well round-bottom plate. Centrifuge for 5 min at 1500 rpm and discard the supernatant.

Resuspend the cells in pre-warmed antibody and probes mix (50 µL per well). Add 50µL of each FMO mix to their respective wells and add 50 µL of PBS + 2% FCS to the unstained well. Incubate for 15 min at 37 °C.

Once the staining time is over, add 100 uL of warm PBS + 2% FCS to wash the mix off the cells and centrifuge again (5 min at 1500 rpm and discard the supernatant).

Finally, resuspend the cells in PBS + 2% FCS for acquisition in the flow cytometer.

Since the MitoTracker Green dye is not fixable, the samples should be acquired as soon as the staining is over to minimize cell death.

### MitoSOX staining: production of mitochondrial superoxide

MitoSOX Red details:

- Stock solution (reconstituted in DMSO): 5 mM
- Working concentration: 5 µM

#### Preparation of mix

Since MitoTracker Red and MitoSOX have very similar excitation and emission wavelengths, these two probes cannot be used together. Therefore, we stained MitoSOX separately, using the panel from Table 2.

**Table 2:** Flow cytometry panel for evaluation of mitochondrial ROS in BMDMs.

Target/Probe	Fluorochrome	Clone/ Cat. number
Live/Dead Calcein	Blue Excitation⁄Emission (nm): 495/515	BD Calcein Blue AM Fluorescent Dye FisherScientific Catalog number: BDB564060
Anti-mouse CD11b	PE-Cy7 Excitation⁄Emission (nm): 496/785	Clone: M1/70 Isotype: Rat IgG2b, κ Biolegend Catalog number: 101,216
MitoSOX™	PE - Red Excitation⁄Emission (nm): 510/580	MitoSOX™ Red Mitochondrial Superoxide Indicator Invitrogen™ Catalog number: M36008

For macrophage surface staining and Live/ Dead staining, we diluted the anti-CD11b antibody 1/200 and the Live/Dead probe was diluted 1/500 in PBS + 2% FCS.

From the stock solution of MitoSOX Red, we suggest diluting 1/10 in PBS and then 1/100 into the final mix. This results in a final working concentration of 1/1000, or 5 µM.

Again, we determine one well for our unstained control and 1 well for MitoSOX Red FMO. The FMO consists of 50 µL of all dyes, except MitoSOX Red.

Pre-warm the mix (37 °C) and stain the cells as described above in the cell staining guide.

## Representative results

### Gating strategy for basic mitochondrial parameters

Mitochondrial function plays an extremely important role in immune cells and in the regulation of the inflammatory response [4]. Here, we describe a method for the study of mitochondrial function that is entirely based on flow cytometry, a tool that is commonly used by immunologists. We used BMDMs and treated them with 100 ng/mL of lipopolysaccharide (LPS) from the membrane of gram-negative bacteria and can activate a series of immune cells, including monocytes, dendritic cells and macrophages to induce an inflammatory response [5].

**Fig. 1:**
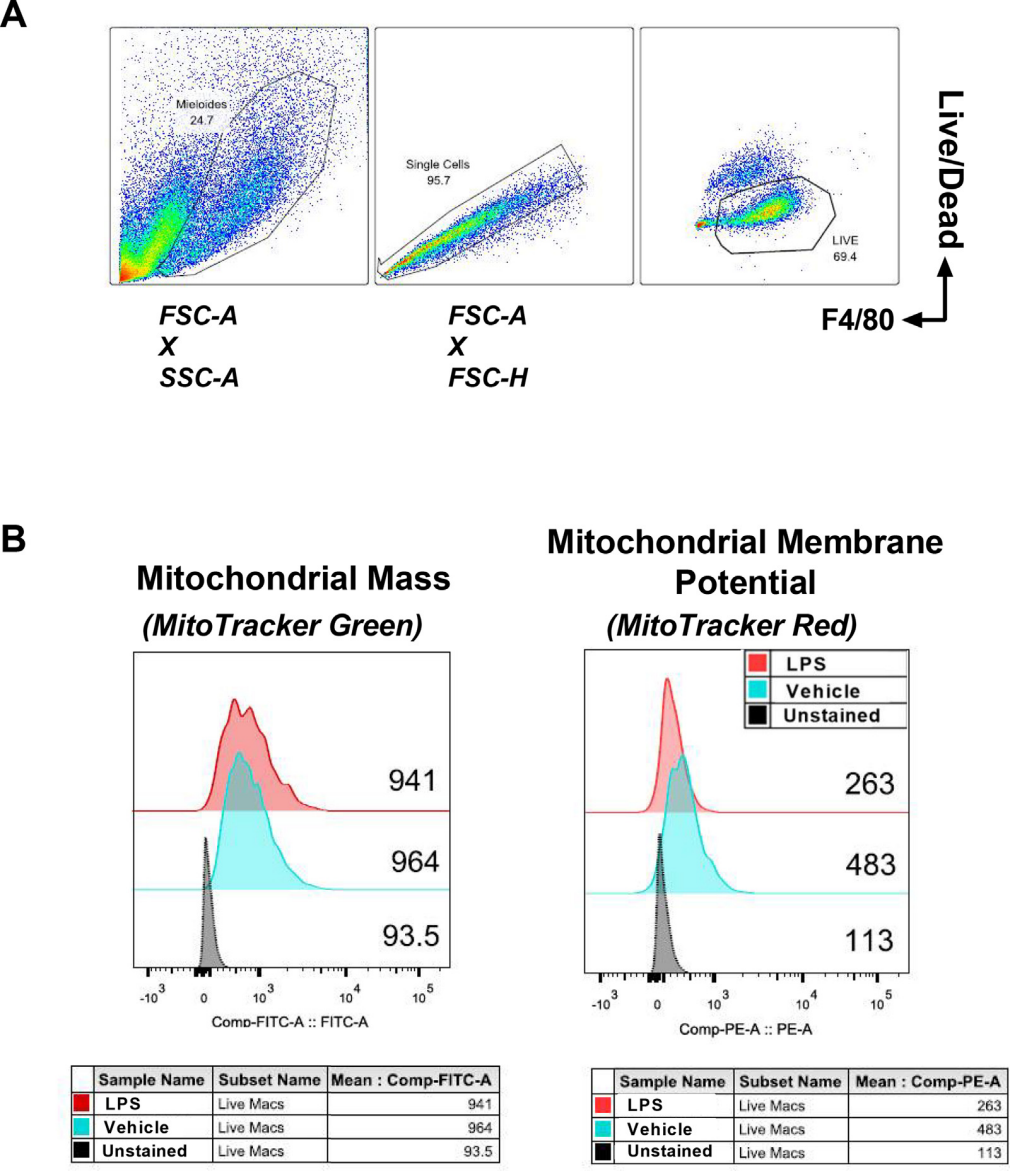
Analysis of mitochondrial mass and membrane potential using flow cytometry probes. BMDMs were treated with 100 ng/mL LPS for 6 h and stained with: macrophage surface marker F4/80, a Live/Dead fluorescent dye and the mitochondrial probes MitoTracker Green and MitoTracker Red. A. Gating strategy to select living macrophages, stained F4/80^+^Live/Dead^−^. B. Representative MFI of mitochondrial mass content in macrophages treated with vehicle (PBS) and LPS. Mitochondrial mass is detected by the MitoTracker Green probe. C. Representative MFI of mitochondrial membrane potential in macrophages treated with vehicle (PBS) and LPS. Mitochondrial membrane potential is shown by the MitoTracker Red probe staining.

We selected live macrophages for our analysis, according to the panel on Table 1 (Fig. 1A). They are identified as Live in the gate of F4/80^+^ and Live/Dead^−^ cells. Since the mitochondrial probe MitoTracker green is not fixable, we often see high amounts of cell death depending on how long it takes to acquire the samples. From our F4/80^+^ Live/Dead^−^ macrophage gate, we opened two distinct histograms in the FITC and PE channels, MitoTracker Green and red MitoTracker Red, respectively (Fig. 1B).

MitoTracker Green is a mitochondrial selective probe that binds covalently to mitochondrial proteins by reacting with cysteine residues and accumulates in the mitochondrial matrix [6]. This reaction is independent of mitochondrial membrane potential and is used to represent mitochondrial mass [7]. We analysed the mean fluorescence intensity (MFI) of vehicle (PBS) and LPS-treated macrophages for MitoTracker Green probe and observed that, even after LPS stimulus, the mitochondrial mass in macrophages remains similar to vehicle-treated cells (Fig. 1B left).

The other probe used in this experiment is the MitoTracker Red. This probe is taken up by polarized mitochondria that are negatively charged [8]. Therefore, it is considered to be dependent on mitochondrial membrane potential (ΔΨ_m_) [9]. Knowing that ΔΨ_m_ changes upon specific stimuli [10], [11], [12], MitoTracker Red can be used to determine gain or loss of mitochondrial functionality [13]. On steady state, macrophages present high mitochondrial polarization. After LPS-induced macrophage activation, we observe a loss in ΔΨ_m,_ through MitoTracker Red MFI (Fig. 1B right). ΔΨ_m_ loss is associated with the metabolic shift from OXPHOS to glycolysis in inflammatory immune cells [13].

### Evaluation of mitochondrial functionality by flow cytometry

**Fig. 2:**
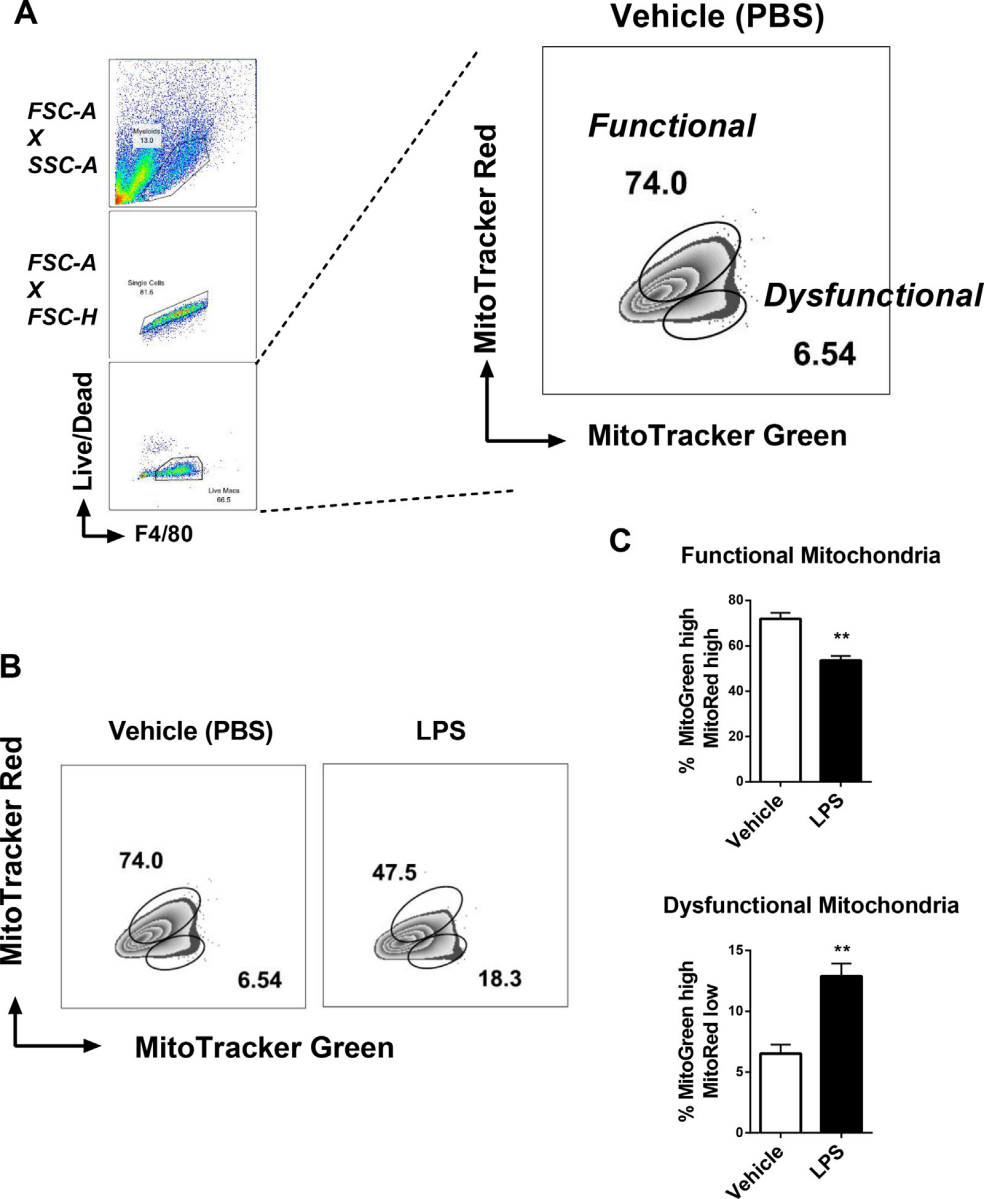
Evaluation of mitochondrial function by flow cytometry. BMDMs were treated with 100 ng/mL LPS for 6 h and stained with: macrophage surface marker F4/80, a Live/Dead fluorescent dye and the mitochondrial probes MitoTracker Green and MitoTracker Red. A. Gating strategy to select cells containing functional mitochondria (MitoTracker Green^high^ and MitoTracker Red^high^) and dysfunctional mitochondria (MitoTracker Green^high^ and MitoTracker Red^low^). B. Representative dotplots of functional and dysfunctional mitochondria in macrophages treated with vehicle (PBS) and LPS. C. Quantification of the percentage of functional and dysfunctional mitochondria in macrophages treated with vehicle (PBS) and LPS. Error bars are mean ± SEM **p < 0.01, (*n* = 4).

In this experiment, we use flow cytometry staining to determine mitochondrial function based on mitochondrial mass and membrane potential. We used the same panel as Fig. 1 and analysed both parameters together (MitoTracker Green x MitoTracker Red) from the gate of living macrophages (F4/80^+^ and Live/Dead^−^) (Fig. 2A). For this type of analysis, we consider functional mitochondria the gate containing cells stained MitoTracker Green^high^ and MitoTracker Red^high^. The gate containing cells stained MitoTracker Green^high^ and Mitotracker Red^low^ represents the percentage of dysfunctional mitochondria, which are mitochondria that suffered loss of membrane potential (Fig. 2A) (14).

When macrophages are stimulated with LPS, they become active and suffer intense metabolic reprogramming, which leads to loss of ΔΨ_m_ and accumulation of MitoTracker Red^low^ staining when compared with vehicle treated macrophages (Fig. 2B). The use of both probes results in a double-gated dot plot containing one region of functional mitochondria and one region of dysfunctional mitochondria (Fig. 2B). The percentage values can be plotted in representative bar graphs for statistical analysis (Fig. 2C). Here, we show the representative graph of 2 independent experiments showing that LPS treatment reduces the percentage of functional mitochondria and leads to the accumulation of dysfunctional mitochondria in macrophages (Fig. 2C). This technique can be used in different cell types and pathological conditions, since many different cells undergo metabolic reprogramming associated with mitochondrial function besides macrophages [15], [16], [17], [18].

### Analysis of mitochondrial ROS production by pro-inflammatory macrophages

During oxidative phosphorylation part of the electrons flowing through the ETC “leaks” and rapidly interacts with molecular oxygen to form superoxide anion, which is the most important ROS in the mitochondria [19]. MitoSox is a live-cell permeant dye with the cationic triphenylphosphonium substituent, the positive charge of this molecule allows the uptake of the probe by actively respiring mitochondria. Once inside of the mitochondria, another constituent of MitoSOX called dihydroethidium can be oxidized by superoxide generating the 2-hydroxyethidium, which exhibits a fluorescence excitation peak at ~400 nm and emission detection at ~590 nm [20].

**Fig. 3:**
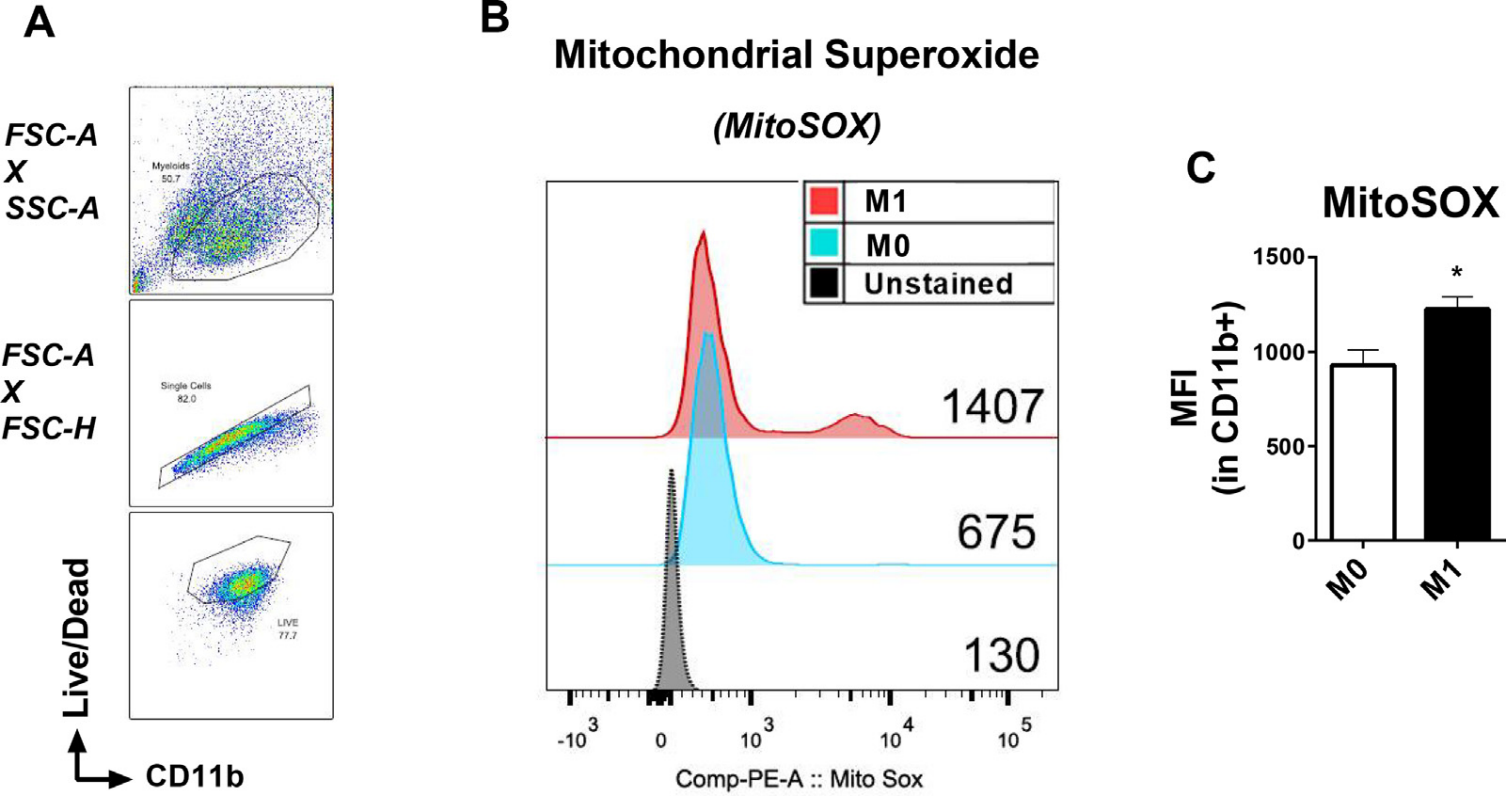
Analysis of mitochondrial superoxide production by M1 macrophages. BMDMs. were treated with 20 ng/m0L IFN- γ + 100 ng/mL LPS for 6 h to induce an M1 phenotype. Macrophages were stained with: macrophage surface marker CD11b, a Live/Dead fluorescent dye and the mitochondrial probe MitoSOX Red. A. Gating strategy to select living macrophages, stained CD11b^+^Live/Dead^+^. B. Representative MFI of mitochondrial superoxide production by macrophages treated with either vehicle (M0) or IFN- γ+LPS (M1). C. Quantified MFI of mitochondrial ROS production by M0 and M1 macrophages. Error bars are mean ± SEM *p < 0.05, (*n* = 6).

Here, we stained the macrophages using the panel described on Table 2. For our living cells selection, we used calcein, which is a cell permeant dye that is hydrolysed by living cells and becomes fluorescent. Therefore, we select the Live/Dead^+^ events. Previous studies have described that M1 macrophages produce more mitochondrial ROS and that is important for the killing of pathogens [21]. Taking this into consideration, we stimulated BMDMs with IFN-γ (20 ng/mL) + LPS (100 ng/mL) to induce an M1 phenotype (classically activated, pro-inflammatory macrophages). The M0 macrophages were given PBS. We selected macrophages from the gate of living cells (Live/Dead^+^ and CD11b^+^) (Fig. 3A). We used a representative histogram to show that the M1 macrophages have higher MitoSOX MFI than M0 macrophages after 6 h of IFN-γ and LPS treatment (Fig. 3B). This result is quantified in a bar graph with statistical analysis (*n* = 6) (Fig. 3C).

## Conclusion

In this work, we describe how mitochondrial specific dyes can be used to track mitochondrial function in a simple flow cytometry assay. The combination of MitoTracker Red and MitoTracker Green provides a simple strategy to analyze how different treatments can change the content of respiratory and non-respiratory mitochondria. Also, the MFI provides an indirect measurement of ΔΨ_m_ and mitochondrial mass. We provide a step by step analysis of mitochondrial ROS production with MitoSOX. This protocol contains a compilation of mitochondrial assays that are suitable for flow cytometry and can be adapted to include as many cell markers as the color panel for the cytometer allows. Altogether, these dyes are a powerful tool for researchers with limited number of cells and provide a general understanding of how mitochondria are altered during different treatments and physiological or pathological states.

## Declaration of Competing Interest

The authors declare no conflict of interest. Graphical abstract was created BioRender, ©biorender.com. The authors declare that they have no known competing financial interests or personal relationships that could have appeared to influence the work reported in this paper.
